# Non-invasive prediction of the tumor growth rate using advanced diffusion models in head and neck squamous cell carcinoma patients

**DOI:** 10.18632/oncotarget.16851

**Published:** 2017-04-05

**Authors:** Noriyuki Fujima, Tomohiro Sakashita, Akihiro Homma, Taisuke Harada, Yukie Shimizu, Khin Khin Tha, Kohsuke Kudo, Hiroki Shirato

**Affiliations:** ^1^ Department of Diagnostic and Interventional Radiology, Hokkaido University Hospital, Sapporo, Japan; ^2^ Department of Otolaryngology, Head and Neck Surgery, Hokkaido University Graduate School of Medicine, Sapporo, Japan; ^3^ Department of Radiation Medicine, Hokkaido University Graduate School of Medicine, Sapporo, Japan; ^4^ The Global Station for Quantum Medical Science and Engineering, Global Institution for Collaborative Research and Education, Sapporo, Japan

**Keywords:** head and neck squamous cell carcinoma, tumor growth rate, magnetic resonance imaging, diffusion weighted imaging, advanced diffusion model

## Abstract

We assessed parameters of advanced diffusion weighted imaging (DWI) models for the prediction of the tumor growth rate in 55 head and neck squamous cell carcinoma (HNSCC) patients. The DWI acquisition used single-shot spin-echo echo-planar imaging with 12 b-values (0−2000). We calculated 14 DWI parameters using mono-exponential, bi-exponential, tri-exponential, stretched exponential and diffusion kurtosis imaging models. We directly measured the tumor growth rate from two sets of different-date imaging data. We divided the patients into a discovery group (*n* = 40) and validation group (*n* = 15) based on their MR acquisition dates. In the discovery group, we performed univariate and multivariate regression analyses to establish the multiple regression equation for the prediction of the tumor growth rate using diffusion parameters. The equation obtained with the discovery group was applied to the validation group for the confirmation of the equation's accuracy. After the univariate and multivariate regression analyses in the discovery-group patients, the estimated tumor growth rate equation was established by using the significant parameters of intermediate diffusion coefficient D_2_ and slow diffusion coefficient D_3_ obtained by the tri-exponential model. The discovery group's correlation coefficient between the estimated and directly measured tumor growth rates was 0.74. In the validation group, the correlation coefficient (*r* = 0.66) and intra-class correlation coefficient (0.65) between the estimated and directly measured tumor growth rates were respectively good. In conclusion, advanced DWI model parameters can be a predictor for determining HNSCC patients’ tumor growth rate.

## INTRODUCTION

The tumor growth rate is an important characteristic for the assessment of numerous types of tumors, including head and neck squamous cell carcinoma (HNSCC). Progressive HNSCCs can show early growth and advanced T-stage, which are directly related to poor patient prognosis [1], and thus a determination of the tumor growth rate would be useful for the determination of treatment priorities and the details of each patient-based treatment plan.

Exact tumor growth rate measurement has been achieved by directly measuring a tumor's doubling time with a comparison of imaging data obtained on two different dates. However, the determination of an HNSCC's growth rate at the pretreatment stage is usually difficult because the details of treatment for an HNSCC must be planned as soon as possible, even before assessing the tumor growth rate. The histopathological evaluation of tumors by immunostaining, such as that revealing an increased Ki-67 index or epidermal growth factor receptor (EGFR) expression, has also been shown to reflect tumor progression and development [2]. However, an evaluation of the total tumor histological findings before the start of treatment would not be possible. Even if a biopsy is performed, such a small tumor tissue sample cannot be assumed to reflect the entire tumor's Ki-67 index or EGFR expression.

Information about a tumor's microstructure is also thought to relate to tumor progression, to a degree. For example, microstructural information such as the cellular density shown by the apparent diffusion coefficient (ADC) obtained by magnetic resonance (MR) diffusion weighted imaging (DWI) was reported to be correlated with the histological Ki-67 index [3]. If a tumor's microenvironment can be revealed in greater detail, such information could well reflect the tumor's aggressiveness. Advanced diffusion models such as a bi-exponential model, a tri-exponential model, a stretched exponential model (SEM) and a diffusion kurtosis imaging (DKI) model have been reported, and they have been used to examine head and neck lesions [4–17]. The parameters obtained from these advanced diffusion models can reflect the tumor tissue microenvironment better than conventional diffusion models that use a mono-exponential model parameter (e.g., the ADC). These models also provide new information related to a tumor's microstructure.

We conducted the present study to assess the microstructure-related information from diffusion parameters obtained by the advanced diffusion models for the prediction of the tumor growth rate in patients with HNSCC.

## RESULTS

The characteristics of the primary lesions of the 55 patients are summarized in Table 1. For all 55 patients, the duration between the 1st and 2nd scans for the tumor growth rate calculation was 34.2 ± 4.15 days (min. 25 days, max. 43 days). Tumor size was measured by CT in 14 patients and by MRI in 26 patients in the discovery group, and by CT in six patients and MRI in nine patients in the validation group. All MR examinations with multi b-point DWI were performed around the time point of the 2nd scanning of the tumor growth rate calculation; the duration between these two scans ranged from 0 to 5 days.

**Table 1:** Patient characteristics (*n* = 55)

	Discovery group (*n* = 40)	Validation Group (*n* = 15)	Total (*n* = 55)
Age			
Range	47-80	49-76	47-80
Median	63	63	63
Average	62.2	63.3	62.5
Gender			
Male	35	13	48
Female	5	2	7
Primary tumor site			
Oral cavity	11	5	16
Oropharynx	12	4	16
Hypopharynx	6	2	8
Nasal cavity	2	0	2
Paranasal sinus	9	4	13
T-stage			
T1	0	0	0
T2	8	3	11
T3	9	5	14
T4a	20	6	26
T4b	3	1	4
N-stage			
N0	20	6	26
N1	7	1	8
N2	13	8	21
N3	0	0	0
Smoking status			
Tobacco smokers	37	13	50
Packs-years			
Range	2-94	10-110	2-110
Median	37	30	34
Average	40.4	42.3	35.2
Alcohol use			
Occasional or non-drinker	8	3	11
Moderate use	15	5	20
Heavy use	17	7	24
Treatment modality			
Surgery	5	4	9
Chemoradiation	34	10	44
Palliative care	1	1	2

The calculation of all parameters in the advanced fitting models was successfully performed. The ROI size used for the diffusion parameter analysis was 9.58 ± 6.89 cm^2^ (min. 3.64 cm^2^, max. 35.52 cm^2^). The tumor size for the tumor growth rate measurement was 7.34 cm^2^ (min. 2.79 cm^2^, max. 20.18 cm^2^) in 1st scan, and 9.79 cm^2^ (min. 3.34 cm^2^, max. 27.81 cm^2^) in 2nd scan. The more details of the measured tumor size in the 1st and 2nd scans, the tumor growth rate, and the diffusion parameters are summarized in Table 2. The measured SNR data in all patients are presented in Supplementary Table 1. There was no significant difference between discovery and validation group patients in patient characteristics, tumor size, tumor growth rate and diffusion parameters (*p* > 0.05).

**Table 2:** Details of diffusion parameters

	Discovery group	Validation group
ADC	0.95 ± 0.15	0.97 ± 0.21
D*	19.8 ± 7.8	20.5 ± 8.2
f	0.16 ± 0.06	0.16 ± 0.07
D	0.74 ± 0.07	0.76 ± 0.1
f_1_	0.12 ± 0.04	0.12 ± 0.04
f_2_	0.24 ± 0.04	0.25 ± 0.04
f_3_	0.64 ± 0.06	0.63 ± 0.06
D_1_	29 ± 9.7	29.9 ± 10.6
D_2_	0.93 ± 0.16	0.94 ± 0.17
D_3_	0.63 ± 0.1	0.62 ± 0.09
α	0.68 ± 0.09	0.66 ± 0.08
DDC	1.06 ± 0.19	1.11 ± 0.22
D_k_	1.19 ± 0.21	1.23 ± 0.24
K	0.78 ± 0.12	0.79 ± 0.11
tumor size in 1st scan	7.26 ± 4.32	7.55 ± 4.58
tumor size in 2nd scan	9.78 ± 6.51	9.81 ± 6.45
tumor growth rate	134.6 ± 20.1	130.5 ± 16.2

Data are mean ± standard deviation. ADC: apparent diffusion coefficient (×10^−3^ mm^2^/s), D: true diffusion coefficient (× 10^−3^ mm^2^/s), f: perfusion fraction (× 10^2^%), D*: fast diffusion coefficient (×10^−3^ mm^2^/s), f_1_: perfusion-related diffusion fraction (× 10^2^%), f_2_: intermediate diffusion fraction (×10^2^%), f_3_: slow diffusion fraction (×10^2^%), D_1_: perfusion-related diffusion coefficient (×10^−3^ mm^2^/s), D_2_: intermediate diffusion coefficient (×10^−3^ mm^2^/s), D_3_: slow diffusion coefficient (×10^−3^ mm^2^/s), α: diffusion heterogeneity (dimensionless), DDC: distributed diffusion coefficient (×10^−3^ mm^2^/s), K: kurtosis value (dimensionless), D_k_: kurtosis corrected diffusion coefficient (×10^−3^ mm^2^/s), tumor size in 1st scan (cm^2^), tumor size in 2st scan (cm^2^), tumor growth rate (%). The directly measured tumor size and its growth rate.

### The holdout validation technique (1): The discovery group

In the univariate regression analysis, the values of the parameters of the ADC, D, D_k_, DDC, D_2_, D_3_, f_2_ and f_3_ showed a significant relationship with the tumor growth rate, whereas none of the patients’ characteristics were significantly related to the tumor growth rate. The results of the univariate regression analysis are presented in Table 3.

**Table 3:** Univariate regression analysis results

	*p*-value	Correlation coefficient
Age	0.28	0.18
Sex	0.91	0.02
Location	0.3	0.16
T-stage	0.13	0.23
N-stage	0.52	0.1
Smoking status	0.51	0.1
Alcohol use	0.9	0.02
ADC	0.0002*	−0.54
D*	0.14	−0.23
f	0.12	−0.25
D	> 0.0001*	−0.61
f_1_	0.48	−0.12
f_2_	0.009*	−0.41
f_3_	0.01*	0.4
D_1_	0.16	−0.22
D_2_	> 0.0001*	−0.59
D_3_	> 0.0001*	−0.65
α	0.09	−0.28
DDC	0.0001*	−0.55
D_k_	0.0001*	−0.56
K	0.06	0.3
Tumor size	0.31	0.15

The ‘correlation coefficient’ is the simple correlation coefficient of each parameter to the directly measured tumor growth rate. Abbreviations are explained in the Table 2 footnote. *p < 0 .05.

In the multivariate regression analysis, we first excluded the parameters of ADC, D, D_k_ and DDC from the final output because multicollinearity to other parameters was detected. The parameters D_2_ and D_3_ were both revealed as respective significant variables for the determination of the tumor growth rate. We determined the regression coefficients of each significant parameter, and the regression equation for the estimation of the tumor growth rate was as follows:

Estimated tumor growth rate = (−43.3*10^3^)*D2 + (−91.2*10^3^)*D3 + (227.5).

**Figure 1:**
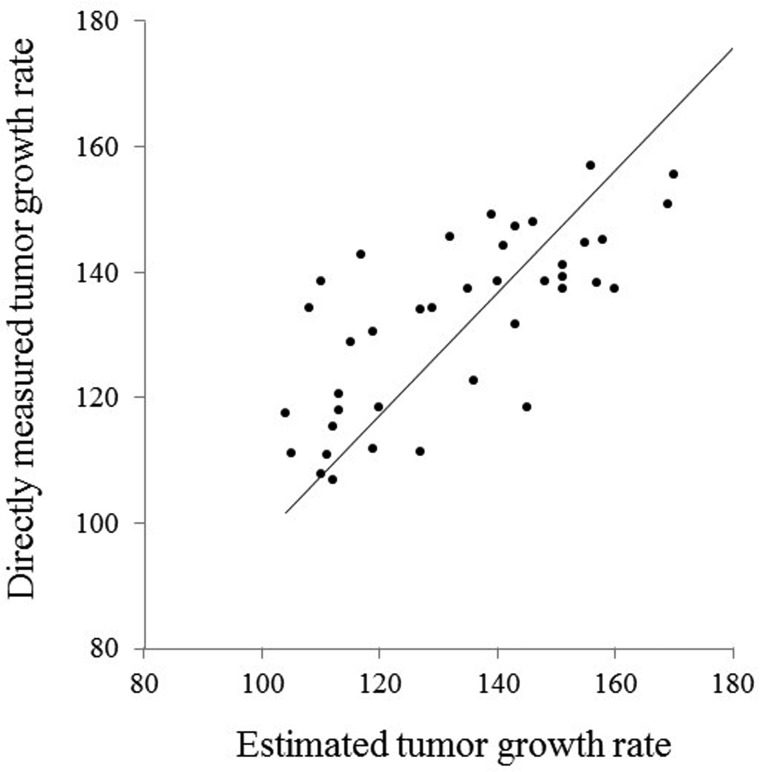
Scatterplot of the estimated and directly measured tumor growth rates in the discovery group patients A good correlation (*r* = 0.74) was observed between the estimated and directly measured tumor growth rates (*p* < 0.001).

The standard partial regression coefficients of D_2_ and D_3_ were −0.34 and −0.45, respectively. The multiple correlation coefficient between the estimated tumor growth rate and the directly measured tumor growth rate in the discovery-group patients was good (0.74). A scatterplot of all of the discovery-group values (the estimated tumor growth rate and the directly measured tumor growth rate) is presented in Figure 1.

### The holdout validation technique (2): The validation group

**Figure 2:**
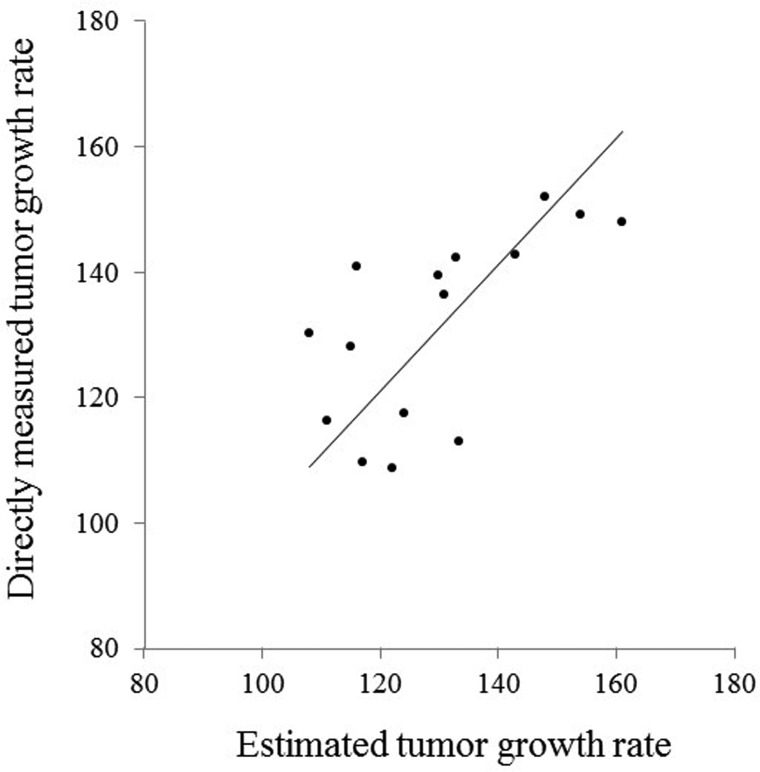
Scatterplot of the estimated and directly measured tumor growth rate in the validation group patients A good correlation (*r* =0.66) and ICC (0.65) were observed between the estimated and directly measured tumor growth rates (*p* < 0.05).

A significant correlation was observed between the estimated tumor growth rate (using the multiple regression equation obtained with the discovery-group data as described above) and the measured tumor growth rate in the validation-group patients (*p* < 0.05). The multiple correlation coefficient (*r* = 0.66) and the ICC (0.65) between the estimated and directly measured tumor growth rates were both good. A scatterplot of all of the validation-group patients is given in Figure 2.

### Five-fold cross validation analysis

The result of each fold and the mean of all five folds revealed mostly the same trend as the results of the holdout validation technique (Supplementary Table 2).

### Inter-observer agreement

The ICC value for each parameter is provided in Table 4. All parameters were revealed as showing excellent interobserver repeatability.

**Table 4:** Intraclass correlation coefficient (ICC) in diffusion parameter measurement between two neruoradiologists

	ICC
ADC	0.98
D*	0.86
f	0.93
D	0.96
f_1_	0.82
f_2_	0.92
f_3_	0.94
D_1_	0.81
D_2_	0.93
D_3_	0.95
α	0.96
DDC	0.96
D_k_	0.97
K	0.97

Data are value of intraclass correlation coefficient (ICC). Other abbreviations are explained in the Table 2 footnote.

### Results of the OS and PFS analyses

**Figure 3:**
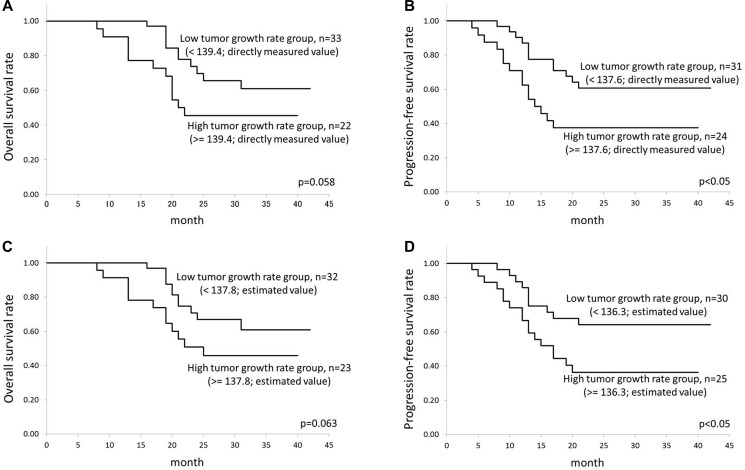
Results of the Kaplan-Meier analysis Results of the Kaplan-Meier analysis for the calculation of: (**A**) OS by the directly measured tumor growth rate, (**B**) PFS by the directly measured tumor growth rate, (**C**) OS by the estimated tumor growth rate, and (**D**) PFS by the estimated tumor growth rate, were presented.

The median follow-up period for the determination of OS was 24 months (range 8–42 months), and that for PFS was 20 months (range 4–42 months). In total 55 patients, in the OS assessment, 32 patients were determined survivors and 23 patients were non-survivors. In addition, in the PFS assessment, 28 patients were determined to have progression-free status and 27 patients were determined not. The cutoff values determined by the ROC curve analysis were as follows: 139.4 (directly measured growth rate in the assessment of OS), 137.6 (directly measured growth rate in the assessment of PFS), 137.8 (estimated growth rate in the assessment of OS), and 136.3 (estimated growth rate in the assessment of PFS). These results indicated that the cutoff values were almost the same between the directly measured and estimated tumor growth rates. In the Kaplan-Meier analysis, the OS rate tended to be better in the low tumor growth rate group, but the difference was not significant (*p* = 0.058 in the directly measured tumor growth rate, *p* = 0.063 in the estimated tumor growth rate). In contrast, the PFS was significantly better in the low tumor growth rate group (*p* < 0.05 in both the directly measured and estimated tumor growth rates). The results of Kaplan-Meier analyses were presented in Figure 3.

## DISCUSSION

Our present findings revealed that in HNSCC patients, the tumor growth rate was related to significantly lower values of D_2_ and D_3_. By using these diffusion parameters, the tumor growth rate can be represented with the multiple regression equation. The tumor growth rate might to be related to the tumor microstructure, because highly progressive tumors will have a rapid cell cycle and high tumor cell proliferation as shown by the histological findings, unlike those of low-progressive tumors [3]. Our present findings suggest that such microstructural differences between tumors with different tumor growth rates can be indirectly detected by using the information about the differences in water diffusion with an advanced diffusion model.

Knowing a tumor's growth rate will useful as an additional diagnostic tool for the determination of treatment priorities, for example by identifying patients who may be approaching inoperable status (even shortly before the scheduled operation date). The operation date of such patients can be adjusted by switching their dates with those of patients whose primary lesion is predicted to develop slowly. The tumor growth parameters can also help predict a patient's treatment outcome as a prognostic factor, because a high progression rate will affect the prognosis [18, 19]. Therefore, the determination of advanced diffusion parameters will provide important information for the assessment of a tumor's growth rate by only a single noninvasive MRI scan. Notably, the PFS was significantly better in the patient group with the lower tumor growth rate compared to the PFS of the patients with the higher tumor growth rate, irrespective of treatment methods. This index may thus be used as a prognostic factor.

In HNSCC patients, there is only a very limited number of reports describing the relationships between tumor aggressiveness and MR-derived parameters. The present study is the first to report the relationships between tumor aggressiveness and advanced diffusion model parameters. Surov et al. reported that a lower ADC was significantly correlated with a higher Ki-67 index [3]. It is likely that higher cellular density depicted as a lower ADC in DWI reflects the tumor microstructure of the numerous growing tumor cells in higher-progressive tumors. Our present analyses revealed that the combination of D_2_ and D_3_ in tri-exponential fitting is an indicator that could be used for the prediction of high tumor growth rates. Using a tri-exponential fitting model for HNSCC, another research group speculated that D_2_ fairly or moderately reflects the diffusion of the segment in the extracellular extravascular space (EES), whereas D_3_ also reflects the cellular component [7]. Based on our present findings, we also speculate that HNSCCs with a high growth rate will have decreased water diffusivity in the EES caused by the strongly elevated tumor cellular density in its microenvironment. Such microstructural characteristics may be well observed by the combination of triexponential model parameters in greater detail compared to the ADC.

In contrast, the significance of f_2_ and f_3_ was revealed by the present study's univariate regression analysis, although the correlation value was not high and no significance was observed in the multivariate regression analysis. If the D_2_ and the D_3_ values reflect the EES and cellular space respectively to a certain degree, as mentioned above, these parameters of f_2_ and f_3_ may also reflect the decreased EES and high cellular density in highly progressive tumors. As a previous report stated that the diffusion coefficient parameter reflected not only the cell density but also other characteristics such as the nuclear area and the nuclear-cytoplasmic ratio [20], not only the cell density or cellular compartment but also the cell shape pattern (such as undifferentiated or poorly differentiated SCC patterns) or the cell size and the cell line complexity may be partially reflected by the diffusion coefficients D_2_ and D_3_ rather than the tissue fractions f_2_ and f_3_. These factors might also reflect the characteristics of highly progressive tumors in greater detail.

In addition, the intermediate diffusion-related coefficients including D_2_, ADC, DDC and D_k_ were each revealed to have a significant relationship with the tumor growth rate in our univariate regression analysis. However, these parameters (except D_2_) were deleted from the final output for the multivariate analysis by multicollinearity. The ranges of these diffusion parameters were all observed to be around 1.0 × 10^−3^ mm/s^2^ in this study. An earlier report also stated that these parameters were well correlated with each other [7]. These parameters might reflect almost the same structure, and thus can be used interchangeably.

Our present findings also revealed that the perfusion-related parameters D*, D_1_, f and f_1_ and the diffusion heterogeneity parameters K and α were not significantly correlated with the tumor growth rate. Tumor perfusion is one of the important characteristics that reflect the tumor biology related to hypoxia [21]. In addition, diffusion heterogeneity parameters may reflect aspects of tissue microstructural complexity such as the membrane structure or the balance of cellular space and EES [22]. Since there is a possibility that these parameters affect the tumor growth rate under certain circumstances, further investigations that include subgroup analyses are needed.

Our study has several limitations. It was a retrospective study, and the patient number was small. The direct measurement of the tumor growth rate in a prospective design was difficult because most patients with HNSCC are advised to start treatment as soon as possible; this underlies the present study's retrospective design and small patient number. Second, the comparison of histological findings was not confirmed. For the assessment of tumor growth or aggressiveness, a histological index such as Ki-67 is a commonly used indicator. However, most patients in this study underwent only a biopsy, and not a tumor total resection. A previous report noted that the Ki-67 index in HNSCC had intratumoral heterogeneity along with a wide range of values [23]. If a small sample obtained by biopsy is assessed for the determination of the Ki-67 index, the level of expression will be affected depending on the area from which the sample was obtained, and we thus suggest that it would be unwise to use such a Ki-67 index (by biopsy tissue only) as the indicator of the entire tumor's aggressiveness. We believe that the direct measurement of the tumor growth rate will well reflect the whole tumor's aggressiveness. Third, our study included both nasal cavity/paranasal sinus tumors and pharynx/oral cavity group tumors. The biological characteristics of these two groups might be somewhat different, and thus a further subgroup analysis is needed. However, the diffusion parameters of these two subtypes were reported to not differ significantly [7]. We also found that the information of the primary site was not significantly associated with the tumor growth rate in a univariate analysis. From this point of view, we speculate that the diffusion parameters can be used for the prediction of tumor growth rate regardless of the difference between the two groups of primary sites (nasal cavity/paranasal sinus and pharynx/oral cavity). Fourth, only DWI parameters with the fixed arrangement of b-value were investigated. A more simple acquisition and calculation method for a shorter scan/analysis time should be established with the best b-value arrangement by comparing parameters between various b-value data. In addition to DWI parameters, it is likely that there are numerous other factors that affect the tumor growth rate. Large cohort analyses assessing multiple factors (including histological information, genetic information and human papilloma-virus status) are needed for the determination of their correlations, along with detailed subgroup analyses.

In conclusion, advanced diffusion model parameters, especially the tri-exponential model parameters, might be predictors for determining the tumor growth rate in HNSCC patients. This information could also help the decision-making regarding treatment options.

## MATERIALS AND METHODS

### Patients

The protocol of this retrospective study was approved by our institutional review board, and written informed consent was waived. We evaluated the cases of 55 patients with HNSCC who were treated at our hospital from January 2012 to November 2015. All patients fulfilled the following inclusion criteria: (1) the patient was first diagnosed (not a recurrent case) histopathologically as having HNSCC; (2) MR imaging (MRI) including multi b-point DWI was performed before any treatment; and (3) two sets of scans by the same modality (either contrast-enhanced computed tomography (CT) or MRI including T1-weighted imaging (T1WI) and fat suppressed (Fs) T2WI) were performed, which enabled the measurement of the tumor growth rate. Although the modality used to determine the tumor size and its growth rate measurement differed between patients (CT was used for some patients and MRI was used for others), a prior study demonstrated that there was no significant difference in the tumor size measured by CT and that obtained by MRI when setting the gross tumor volume for HNSCC [24], and we thus considered that a problematic difference in tumor size measurement by CT versus MRI would not have occurred. Only cases in which the same slice angle was used for the acquisition between the two scans were selected for this study population. Patients who underwent a biopsy during the period from three weeks before the 1st scan to the 2nd scan were excluded from the study, because the influence of a biopsy (i.e., tissue inflammation) around the tumor will disturb the correct tumor size measurement. Patients with a severe metal artifact (which can seriously affect the image quality of the primary lesion) and patients with a primary site in the nasopharynx or salivary gland were also excluded. Human papilloma-virus status was reported to be one of the important biological characteristics that can be used as a prognostic factor in HNSCC patients, especially those with oropharyngeal cancer [25]. However, over the duration of the present study, an analysis of the Human papilloma-virus status was not routinely performed at our hospital, and most of the present study's population had not undergone this analysis. We therefore could not include the human papilloma-virus status information as part of the patient data. We divided the final total of 55 patients into the discovery group (40 patients) and the validation group (15 patients) based on their MR acquisition dates (older dates; discovery group, more recent dates; validation group).

### Imaging protocol

All MR imaging was performed using a 3.0 Tesla unit (Achieva TX; Philips Healthcare, Best, Netherlands) with a 16-channel neurovascular coil. The DWI acquisition used single-shot spin-echo echo-planar imaging (EPI) with three orthogonal motion probing gradients. Twelve b-values (0, 10, 20, 30, 50, 80, 100, 200, 400, 800, 1000, and 2000 s/mm^2^) were used. The other imaging parameters were: TR, 4500 msec; TE, 64 msec; DELTA (large delta; gradient time interval), 30.1 msec; delta (small delta; gradient duration), 24.3 msec; flip angle, 90°; field of view (FOV), 230 × 230 mm; 64 × 64 matrix; slice thickness, 5 mm × 20 slices; voxel size 3.59×3.59×5.00 mm; parallel imaging acceleration factor, 2; EPI factor 32; number of signal averages, b-value of 0–100 s/mm^2^ (one average), 200–800 s/mm^2^ (two averages) and 1000–2000 s/mm^2^ (three averages); scanning time, 4 min 37 sec. In this sequence, for the reduction of image distortion in EPI, low in-plane matrix combined with the parallel imaging technique were applied to decrease the number of EPI factor as possible, as described above.

Conventional MRI was also performed to evaluate the primary tumor lesions. These images included (a) an axial T1-weighted image with a spin-echo sequence (TR, 450 msec; TE, 10 msec; FOV, 240 × 240 mm; 512 × 512 matrix; slice thickness, 5 mm; inter-slice gap, 30%; scanning time, 2 min 12 sec) and (b) an axial T2-weighted image (T2WI) with a turbo spin-echo (TSE) sequence with fat suppression (TR, 4500 msec; TE, 70 msec; TSE factor, 9; FOV, 240 × 240 mm; 512 × 512 matrix; slice thickness, 5 mm; inter-slice gap, 30%; scanning time, 2 min 6 sec).

### Data analysis

#### ROI setting

**Figure 4:**
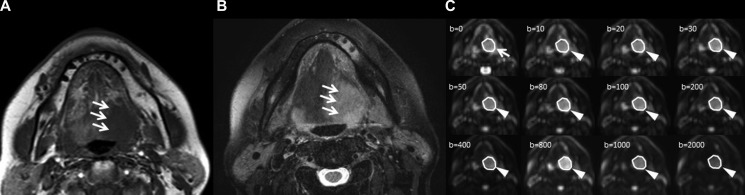
Tumor ROI delineation A patient whose primary tumor was in the base of the tongue is presented. With the depiction of the primary tumor in T1WI (**A**; arrow) and T2WI (**B**; arrow) as a reference for the ROI delineation, the primary tumor was outlined by a polygonal ROI on b0 images of DWI (**C**; arrow), and the tumor ROI was then copied on the EPI of the respective b-values (c; arrowhead). The original images are presented with the same window level/width in the range of 0–400 of b-value, whereas each window level/width was adjusted for good visualization of the tumor for the b-values ≥ 800.

A board-certified neuroradiologist with 19 years of experience delineated each tumor with a polygonal region of interest (ROI) on b0 images; the axial T1WI and T2WI were used as reference images, and the tumor ROI was then copied on the EPI of the respective b-values (Figure 4). Any area which was suspected of being necrosis or a cystic lesion was excluded from the ROI. If the tumor extended into two or more slices, the slice in which the largest area of tumor was depicted was selected. The size of each tumor ROI was calculated.

### Diffusion data calculation

First, the image quality was assessed in all b-value images by determining the signal-to-noise ratio (SNR), which was calculated using the ratio between the mean signal intensities in each tumor ROI (SI_ROI_) and the standard deviation of the background noise (SD_Noise_) (SNR = SI_ROI_ / SD_Noise_).

From the diffusion signal data, we calculated each parameter of mono-exponential function (the apparent diffusion coefficient; ADC), bi-exponential function (the perfusion fraction f, the pseudo-diffusion coefficient D*, and the true diffusion coefficient D), the tri-exponential function (the perfusion-related diffusion fraction f_1_ and coefficient D_1_, the intermediate diffusion fraction f_2_ and coefficient D_2_, and the slow diffusion fraction f_3_ and coefficient D_3_), the SEM (diffusion heterogeneity α and distributed diffusion coefficient DDC) and the DKI (kurtosis value K and kurtosis corrected diffusion coefficient D_k_). Using the signal intensity of all 12 b-values, we calculated the bi-exponential and tri-exponential function parameters. Assessments of the ADC, SEM and DKI usually target the tissue diffusion (except for the perfusion-related signal), and we therefore used the signal intensity of six b-values (0, 200, 400, 800, 1000 and 2000 s/mm^2^) for the parameter calculations of ADC, DKI and SEM. To perform these parameter calculations, we used the following equations [7]:

$$\frac{S_{\left(b\right)}}{S_{0}}=e^{-b\cdot \text{ADC}}$$ (1)

$$\frac{S_{\left(b\right)}}{S_{0}}=f\cdot e^{-b\cdot D*}+(1-f)\cdot e^{-b\cdot D}$$ (2)

$$\frac{S_{\left(b\right)}}{S_{0}}=f_{1}\cdot e^{-b\cdot D_{1}}+f_{2}\cdot e^{-b\cdot D_{2}}+f_{3}\cdot e^{-b\cdot D_{3}}$$ (3)

$$\frac{S_{\left(b\right)}}{S_{0}}=\exp\left[-b*D_{k}+\frac{1}{6}*b^{2}*D_{k}^{2}*K\right]$$ (4)

$$\frac{S_{\left(b\right)}}{S_{0}}=e^{-{\left(b\cdot \text{DDC}\right)}^{\alpha }}$$ (5)

where *S*_(b)_ is the signal intensity at the b-value denoted by the subscript, *S*_0_ is the signal intensity at the b-value of 0, and *b* is b-factor, in all five equations. In the tri-exponential fitting of Eq. (3), the sum of the three parameters f_1_, f_2_ and f_3_ became 1. We fitted the signal intensity of b-values in Eqs. (1–5) with least-square fitting using the Levenberg-Marquardt algorithm. The DW model parameter calculation was performed by the ROI-based approach for the analysis because the calculation process can be performed with a high SNR [26]. In the ROI-based approach, the parameter calculations were performed by using the mean value of each ROI in the multi b-value EPI as the image signal intensity for each b-value. To improve the fitting accuracy and to prevent overfitting in all b-value fitting analyses of the bi-exponential and tri-exponential models, we performed the fitting procedure by the following methods. In the bi-exponential analysis, first, the data of b >200 s/mm^2^ were fitted for the single parameter D by the mono-exponential function. In the second step, the curve was fitted for f and D* over all b-values by using Eq. (2), while keeping D constant. The details of this method were reported [26]. In the tri-exponential analysis, three-step fitting was performed as follows. We first performed mono-exponential fitting with large b-values (800, 1000 and 2000 s/mm^2^) to obtain D_3_, followed by bi-exponential fitting with b-values ≥ 200 s/mm^2^ to obtain D_2_. We next performed tri-exponential fitting by using Eq. (3) with all b-values to obtain D_1_, f_1_, f_2_ and f_3_. The details of this method are also reported [27, 28]. Finally, each parameter in each tumor was calculated.

For the inter-observer reliability assessment of the diffusion parameter calculations, another neuroradiologist with 13 years of experience also delineated each tumor ROI per the above-mentioned method, and then the diffusion parameter calculation was similarly performed. All calculations were performed using MATLAB ver. 2012a (MathWorks, Natick, MA).

### Evaluation of tumor growth rate

Two sets of scans of the same modality (either of contrast-enhanced CT or MRI including T1WI and Fs-T2WI) were used for the tumor progression rate measurement. In the two different-date scans, the area of the tumor in the first scan was measured on the slice in which the largest area of the tumor was depicted. The second scan was performed using the same-level slice at that used for the first scan, which was determined referring to the anatomical information. The tumor growth rate was calculated as the growth rate of each tumor over the interval of 30 days, using the following equation:

$${\text{Size}}_{\text{post}}={\text{Size}}_{\text{pre}}*{\left(\text{Growth}\right)}^{\left(\text{duration}/30\right)}$$

where *Size_pre_* and *Size_post_* are the tumor size at the first and second scans, respectively, *Growth* is the tumor growth rate over the 30-day period (= the estimated tumor size in the 30-day period after the first scan when the tumor size at the first scan is defined as 100), and *duration* is the number of days between the two scans. Thus, we determined the tumor growth rate by using the directly obtained data of tumor size in the two scans and the length of time between the two scans.

### Statistical analyses

Before the Analysis of the holdout validation technique (mentioned below), the Mann-Whitney *U-test* was used to compare patient characteristics, measured tumor size, tumor growth rate and diffusion parameters between discovery and validation group patients.

### Analysis of the holdout validation technique: (1) The discovery group

We first performed a univariate regression analysis to determine the relationship between the tumor growth rate and the patient characteristics, tumor size, and each diffusion parameter. The patient characteristics of age, sex, smoking status, degree of alcohol use, location of the primary site (nasal cavity/paranasal sinus vs. pharynx/oral cavity), T-stage (1, 2, 3 and 4), N-stage (1, 2 and 3), tumor size at the time point of the first scanning and all diffusion parameters were assessed. For each parameter, a regression equation was calculated, and the simple correlation coefficient between the estimated tumor growth rate in the univariate regression equation and the directly measured tumor growth rate was calculated.

As the next step, we performed a multivariate regression analysis to obtain the multiple regression equation for the estimation of the tumor growth rate. Parameters with a significant relationship in the univariate regression analysis were used in the multivariate analysis. Multicollinearity between diffusion parameters was assessed in advance with the variance inflation factors (VIF) (reference value of 10) before the final output to the multivariate regression analysis. Determined parameters without multicollinearity were evaluated by a multivariate regression analysis to clarify which parameters had a significant relation to the tumor growth rate. Last, the multiple regression equation for the estimation of the tumor growth rate was calculated by using the obtained significant variables. The multiple correlation coefficient was calculated for the assessment between the estimated tumor growth rate and the directly measured tumor growth rate.

### Analysis of the holdout validation technique: (2) The validation group

For each tumor, we calculated the estimated tumor growth rate by using the multiple regression equation obtained with the discovery group patients’ data, and we compared the estimated tumor growth rate to the directly measured tumor growth rate. The multiple correlation coefficient and intraclass correlation coefficient (ICC) were determined by comparing the estimated and measured tumor growth rates.

### Cross validation analysis

We also performed a five-fold cross-validation as another validation of each patient characteristic, tumor size, and diffusion parameter analysis. In each fold of the training and test sets, the same evaluation of the discovery and validation groups in the holdout validation technique described above was performed for each group. The patient number of each fold was as follows: training set = 44 and test set = 11. Patients were divided into the five folds randomly.

### Inter-observer agreement

The inter-observer agreement between two neuroradiologists was assessed by determining the ICC in each diffusion parameter.

### Overall survival (OS) and progression-free survival (PFS) analysis

The OS and PFS rates in the total patients (irrespective of treatment methods) were calculated by a Kaplan-Meier curve analysis. In this analysis, we used the log-rank test to compare the OS and PFS between the patient groups with high and low tumor growth rates. The patient groups with high versus low tumor growth rates were divided using the optimal cutoff value, which was determined by conducting a receiver operating characteristic (ROC) analysis. The cutoff value was determined by using the closest point to the upper left corner of the ROC curve in the division of (1) the survivors and non-survivors in the follow-up period for the OS analysis, and (2) the patients with and without progression-free status in the follow-up period for the PFS analysis. The cutoff values were respectively calculated for both the directly measured and estimated tumor growth rates.

All correlation coefficients and ICCs were classified as follows: *r* < 0.2, poor; *r* = 0.2–0.4, fair; *r* = 0.41–0.6, moderate; *r* = 0.61–0.8, good; *r* >0.81, excellent. *P*-values < 0.05 were considered significant. SPSS software (IBM, Armonk, NY) was used for all statistical analyses.

## SUPPLEMENTARY MATERIALS TABLES



## Abbreviations

ADC: apparent diffusion coefficient
CT: computed tomography
D: true diffusion coefficient
D_1_: perfusion-related diffusion coefficient
D_2_: intermediate diffusion coefficient
D_3_: slow diffusion coefficient
D*: fast diffusion coefficient
DDC: distributed diffusion coefficient
D_k_: kurtosis corrected diffusion coefficient
DKI: diffusion kurtosis imaging
DWI: diffusion weighted imaging
EES: extracellular extravascular space
EGFR: epidermal growth factor receptor
EPI: echo-planar imaging
f: perfusion fraction
f_1_: perfusion-related diffusion fraction
f_2_: intermediate diffusion fraction
f_3_: slow diffusion fraction
FOV: field of view
Fs: fat suppressed
HNSCC: head and neck squamous cell carcinoma
ICC: intraclass correlation coefficient
K: kurtosis value
MRI: magnetic resonance imaging
OS: overall survival
PFS: progression-free survival
ROI: region of interest
SEM: stretched exponential model
T1WI: T1-weighted imaging
TSE: spin-echo
VIF: variance inflation factors
α: diffusion heterogeneity
