# Supplementary material for: Non-invasive prediction of the tumor growth rate using advanced diffusion models in head and neck squamous cell carcinoma patients
Source: Oncotarget. 2017 Apr 5;8(20):33631–43. doi: 10.18632/oncotarget.16851 (PMC5464896; doi:10.18632/oncotarget.16851)
Supplement: Supplementary file 1 [file oncotarget-08-33631-s001.pdf]

Non-invasive prediction of the tumor growth rate using advanced diffusion models in head and neck squamous cell carcinoma patients

Supplementary Materials

Supplementary Table 1: Details of signal to noise ratio (SNR) in all b-value images

b-value	SNR
0	94.4 ± 32.2
10	87.5 ± 28.7
20	88.3 ± 30.1
30	84.5 ± 27.6
50	82.1 ± 27.1
80	74.5 ± 26.8
100	70.6 ± 27.3
200	75.2 ± 24.7
400	68.3 ± 23.5
800	53.5 ± 20.7
1000	58.2 ± 20.4
2000	45.4 ± 15.2

Data are mean ± standard deviation. SNR: signal to noise ratio (dimensionless).

Supplementary Table 2: The result of five-fold cross validation analysis

	training set (*n* = 44)			test set (*n* = 11)	
	Univariate significant parameters	Final output parameters	Correlation coefficient	Correlation coefficient	ICC
1st fold	ADC, D, D_k, DDC, D_2, D_3, f_2, f_3	D_2, D_3	0.74	0.7	0.69
2nd fold	ADC, D, D_k, DDC, D_2, D_3, f_3	D_2, D_3	0.76	0.69	0.66
3rd fold	ADC, D, D_k, DDC, D_2, D_3, f_2, f_3	DDC, D_3	0.71	0.63	0.61
4th fold	ADC, D, D_k, DDC, D_2, D_3, f_2, f_3	D_2, D_3	0.72	0.66	0.64
5th fold	ADC, D, D_k, DDC, D_2, D_3, f_3	D_2, D_3	0.77	0.72	0.68
Average			0.74	0.68	0.66

ICC: intraclass correlation coefficient. Abbreviations in ‘Univariate significant parameters’ are explained in the Table 2 footnote.
